# Hydroxytyrosol Modulates Arachidonic Acid Metabolism and Purine Catabolism in Individuals with Prediabetes: An Untargeted Metabolomics Study in a Randomized Controlled Trial

**DOI:** 10.3390/antiox15030317

**Published:** 2026-03-03

**Authors:** Ignacio Moratilla-Rivera, Elisa Fernández-Millán, Jara Pérez-Jiménez, Sonia Ramos, Óscar Yanes, Jordi Capellades, Raquel Mateos, María Ángeles Martín

**Affiliations:** 1 Department of Metabolism and Nutrition, Institute of Food Science, Technology and Nutrition (ICTAN), Consejo Superior de Investigaciones Científicas (CSIC), C/Jose Antonio Novais 6, 28040 Madrid, Spain; i.moratilla@ictan.csic.es (I.M.-R.); jara.perez@ictan.csic.es (J.P.-J.); s.ramos@ictan.csic.es (S.R.); raquel.mateos@ictan.csic.es (R.M.); 2 Faculty of Biological Sciences, Universidad Complutense de Madrid, Ciudad Universitaria s/n, 28040 Madrid, Spain; 3 Departament of Biochemistry and Molecular Biology, Faculty of Pharmacy, Universidad Complutense de Madrid, Plaza de Ramón y Cajal, s/n, 28040 Madrid, Spain; elfernan@ucm.es; 4 CIBER de Diabetes y Enfermedades Metabólicas Asociadas (CIBERDEM), Instituto de Salud Carlos III (ISCIII), 28029 Madrid, Spain; oscar.yanes@urv.cat (Ó.Y.); jordi.capellades@iispv.cat (J.C.); 5 Department of Electronic Engineering, Universitat Rovira i Virgili, Av. dels Països Catalans, 26, 4300 Tarragona, Spain; 6 Metabolomics Platform, Institut de Recerca Biomèdica Catalunya Sud (IRBCatSud), Hospital Universitari Sant Joan de Reus, 43204 Reus, Spain

**Keywords:** hydroxytyrosol, prediabetes, metabolomics, arachidonic acid pathway, purine metabolism

## Abstract

**Background:** Hydroxytyrosol (HT) is a phenolic compound found in extra virgin olive oil that modulates oxidative and inflammatory status. However, clinical trials evaluating HT as a stand-alone supplement remain scarce, and its underlying mechanisms and pathway modulation are not yet fully understood. This study aimed to investigate the metabolic effects of HT supplementation in individuals with overweight and prediabetes using an untargeted metabolomics approach. **Methods:** An untargeted liquid chromatography–mass spectrometry (LC–MS)-based metabolomics analysis was performed on serum samples from 49 participants with overweight and prediabetes enrolled in a randomized controlled trial. Participants received either HT (15 mg/day for 16 weeks; n = 24) or placebo (n = 25). Global metabolomic profiling was used to compare metabolic changes between the two groups. **Results:** HT supplementation induced a distinct metabolic profile compared with placebo. Participants in the HT group showed reduced levels of nitrogenous base derivatives and arachidonic acid, together with increased concentrations of phosphatidylcholines, lysophosphatidylcholines and sphingomyelins. These alterations suggest modulation of two key metabolic pathways including purine degradation and arachidonic acid metabolism. **Conclusions**: These findings provide mechanistic insights into the biological effects of HT and support the integration of metabolomics and multi-omics approaches in future clinical studies to validate these pathways in larger populations.

## 1. Introduction

Type 2 diabetes (T2D) is a metabolic disease that poses a substantial global socioeconomic and healthcare burden, affecting an estimated 589 million adults and causing 3.4 million deaths in 2024, with its prevalence projected to rise to 853 million by 2050 [1]. The strongest risk factors for progression to T2D are excess body adiposity and prediabetes, which are accompanied by oxidative stress, inflammation, and insulin resistance, leading towards a fully established T2D and macrovascular and microvascular damage [2,3]. Therefore, reversion or remission of these factors constitutes a strategy to prevent T2D and its associated complications. In this context, diets rich in bioactive compounds, such as the Mediterranean diet, represent a cost-effective strategy for preventing metabolic diseases. Specifically, extra virgin olive oil has demonstrated protective effects against non-communicable diseases in clinical studies [4,5], which is largely due to its composition, particularly the main phenolic compound, hydroxytyrosol (HT) and its derivatives [6]. However, important gaps remain in clinical trials regarding the mechanisms of action of this phenolic compound.

In recent years, untargeted metabolomics approaches have been increasingly used to explore metabolic alterations associated with diabetes and to assess responses to nutritional interventions. Within this framework, metabolomic approaches have been extensively applied to investigate metabolic changes induced by virgin olive oil, as reported in recent reviews [7,8]. Despite the heterogeneity, these studies have consistently identified several target metabolic pathways, such as glycolysis, the tricarboxylic acid cycle, and amino acid metabolism, that may contribute to the health benefits associated with the intake of this oil [9]. However, the complex composition of virgin olive oil makes it difficult to elucidate the specific contribution of an individual bioactive compound. In the case of HT stand-alone, metabolomic studies specifically assessing the effects of its supplementation remain relatively scarce.

In particular, only two metabolomic studies have investigated HT to date [10,11]. However, one of these did not provide a clear interpretation of the observed metabolic changes [10] and the other one did identify differences in nitrogenous bases and glutathione metabolism between responders and non-responders to HT, but the assays included only six participants [11]. Moreover, none of these studies was focused on individuals with prediabetes. Consequently, the effects of HT supplementation still need to be investigated in clinical trials with a larger sample size, and specifically, in individuals at high T2D risk to further elucidate the underlying mechanisms of action.

Accordingly, our research group previously conducted a clinical trial in forty-nine participants with overweight and prediabetes receiving HT and demonstrated that this phenolic compound may improve oxidative status and inflammation outside its original food matrix [12]. To further elucidate the bioactivity of HT, the present study aimed to investigate the potential metabolic changes associated with its intake. To this end, an LC-MS-based untargeted metabolomics approach was applied to compare the metabolic profiles of participants administered with 15 mg/day of HT for 16 weeks with those receiving placebo, with the objective of identifying differentially expressed metabolites and gaining deeper insight into the biological mechanisms underlying the effects of this natural phenol.

## 2. Materials and Methods

### 2.1. Study Design and Serum Sample Collection

This study was conducted within the framework of a previous investigation into the potential of HT supplementation to prevent age-related diseases in overweight individuals with prediabetes [12]. The study was registered with the International Standard Randomised Controlled Trial Registry (ClinicalTrials.gov; NCT06295913, https://clinicaltrials.gov/study/NCT06295913?intr=Hydroxytyrosol&page=2&rank=14, registered on 20 February 2024).

Sample size was calculated based on oxLDL as the primary endpoint, considering a 30% reduction as clinically relevant, with 95% power and α = 0.05. Based on variance estimates from a previous HT clinical trial [13], 20 participants per group were required; this was increased to 25 per arm to account for a 20% expected dropout, resulting in a total sample of 50 subjects.

Briefly, a randomized, double-blind, placebo-controlled, parallel clinical trial was conducted, involving a total of 52 men and women. The participants were recruited between December 2023 and March 2024 and randomly allocated with a ratio 1:1 to one of two groups by researchers: 26 subjects received an extract with a high HT content (15 mg per day), while 26 subjects received a placebo. At the end, 49 participants finished the study, three withdrew the study, two due to personal reasons and one due to antibiotic treatment. The method used for random allocation was simple randomization with a number generator in Microsoft Excel 2019 MSO and allocation concealment was ensured by storing the randomization sequence in a password-protected file accessible only to authorized personnel not involved in recruitment. Both interventions were administered for 16 weeks.

Eligible participants were overweight adults (body mass index or BMI between 25.0 and 30.0 kg/m^2^) aged 40–70 years with prediabetes, defined by HbA1c values of 5.7–6.4% and/or fasting plasma glucose levels between 100 and 126 mg/dL. Exclusion criteria included the presence of any cardiometabolic disease, hypertension, hyperlipidaemia, hypercholesterolaemia, inflammatory conditions, positive HIV status, current pharmacological treatment, use of other nutritional supplements, smoking, alcohol consumption, pregnancy or lactation. The intervention consisted of consuming one capsule daily with a meal. Placebo capsules containing inert excipients were manufactured to maintain blinding. Capsules in both study arms were identical in appearance, odorless, and indistinguishable to participants and investigators.

Compliance was assessed by measuring urinary hydroxytyrosol-3′-sulfate levels. During the intervention period, participants were instructed to avoid dietary sources of HT, such as extra virgin olive oil, virgin olive oil and olives. To ensure this, olive oil was provided to all participants.

For the metabolomic analysis, fasting blood samples were collected at the beginning and at the end of the study. The samples were centrifuged after collection at 1510× *g* for 10 min at 4 °C, after which the serum as separated and stored at −80 °C until further analysis.

The study was approved by the Ethics Committees of both Hospital Universitario Puerta de Hierro-Majadahonda (Madrid, Spain) and the Spanish National Research Council (CSIC). The study was conducted at the Human Nutrition Unit (HNU) of the Institute of Food Science, Technology and Nutrition (ICTAN-CSIC, Madrid, Spain) between March 2024 and September 2024. Written informed consent was obtained from all participants prior to their inclusion in the study and were instructed to report any adverse events experienced during the intervention period.

### 2.2. LC-MS Methods

#### 2.2.1. Metabolite Extraction

Serum metabolites were extracted by mixing 25 μL of serum and 300 μL of cold acetonitrile:methanol:water (5:4:1/*v*:*v*:*v*). The samples were then kept in ice for 30 min before being centrifuged at 24,149× *g* for 10 min at 4 °C. Quality control (QC) samples were prepared in the same way by pooling aliquots from each participant and study visit. The resulting supernatant was transferred to LC/MS-compatible vial for analysis.

#### 2.2.2. LC-MS Settings

Serum samples were analyzed using an Orbitrap IDX Tribrid mass spectrometer (Thermo Scientific, Waltham, MA, USA), which was equipped with a heated electrospray ionization (HESI) source and coupled to a Vanquish Ultra-High-Performance Liquid Chromatography (UHP-LC) system. Chromatographic separation was performed using an Acquity UPLC BEH HILIC column (2.1 × 150 mm, 1.7 µm; Waters, Milford, MA, USA). Mobile phase A consisted of acetonitrile containing 50 mM ammonium acetate, while mobile phase B consisted of 50 mM ammonium acetate in water. A gradient elution was applied at a flow rate of 0.40 mL/min, using the following optimized program: 5% B held isocratically for 2 min, increased to 50% B over 4 min, and maintained for 1 min. The system was then returned to initial conditions within 0.2 min and re-equilibrated for 3.2 min. The total run time was 10.5 min. The column temperature was maintained at 25 °C, and the injection volume was 5 μL. QC samples were injected at the beginning of the analytical run and at regular intervals throughout the chromatographic sequence.

Mass spectrometric detection was performed in both negative and positive ionization modes using the following HESI source parameters: spray voltage, 2.8 kV (negative) and 3.5 kV (positive); ion transfer tube temperature, 300 °C; vaporizer temperature, 300 °C; sheath gas (N_2_) flow rate, 50 a.u.; auxiliary gas (N_2_) flow rate, 10 a.u.; sweep gas (N_2_) flow rate, 1 a.u.; S-lens RF level, 60%. Data were acquired in full-scan mode with a resolution of 120,000 (at *m/z* 200), an AGC target of 50%, and a maximum injection time of 200 ms.

MS/MS acquisition was performed at a resolution of 15,000 using higher-energy collisional dissociation (HCD) with a normalized collision energy mixing energies 10-20-30-40%, which was optimized to obtain adequate precursor and product ion intensities. The quadrupole isolation window was set to 1 *m*/*z*. The maximum C-trap injection time was customized for each inclusion list. Instrument control and data acquisition were carried out using Xcalibur software version 4.4 (Thermo Scientific, Waltham, MA, USA).

#### 2.2.3. Metabolite Identification by MS/MS Spectra

Metabolite identification was performed using HERMES software through two complementary strategies: (i) cosine spectral matching against an in-house database containing MS/MS spectra from the MassBankEU, MoNA, HMDB, RIKEN, and NIST14 databases, and (ii) spectral matching using MassFrontier version 8.0 SR1 (Thermo Scientific, Waltham, MA, USA) against the mzCloud database. Spectral matches with high similarity scores (>0.8) were manually reviewed to ensure accurate metabolite identification.

### 2.3. Statistical Analysis

Baseline characteristics were analyzed to assess the presence of significant intergroup differences in age, sex, body mass index (BMI), HbA1c, and fasting glucose levels. Quantitative variables were compared using the unpaired Student’s *t*-test, while sex distribution was evaluated using the chi-squared (χ^2^) test. Statistical significance was set at *p* < 0.05.

LC/MS data were processed using the HERMES R package [14] for MS1 profiling, quantification, and inclusion list generation for metabolite identification. The analysis was performed using a database containing 22,314 unique molecular formulas from ChEBI and HMDB.

Statistical analyses were performed on the log_2_-transformed final-to-initial concentration ratios [log_2_(FC/IC)] for each metabolite to identify those showing the most pronounced changes between intervention groups. Only signals of interest (SOI) quantified in more than 80% of samples were included in the statistical testing. Group differences were assessed using the unpaired Student’s *t*-test. All samples of the 49 participants pre- and post-intervention were included for the analysis.

A multivariate analysis of the log_2_(FC/IC) data was conducted using the MetaboAnalyst 6.0 online platform, applying total ion current normalization and Pareto scaling. This yielded the following: a Principal Component Analysis (PCA), a heatmap with hierarchical clustering based on the intervention, and a Partial Least Squares Discriminant Analysis (PLS-DA) to identify the Top 10 important features.

Finally, the correlations between the log_2_(FC/IC) values of the metabolites and the oxidative stress and inflammation parameters (the latter published in [12]) were assessed using Pearson’s correlation coefficient. One participant with several undetermined parameters was excluded for the analysis. The results were visualized as a heatmap, where color intensity reflects the strength and direction of the correlation; red indicates stronger positive correlation, while blue indicates stronger negative correlation.

## 3. Results

### 3.1. Participant Characteristics

**Figure 1:**
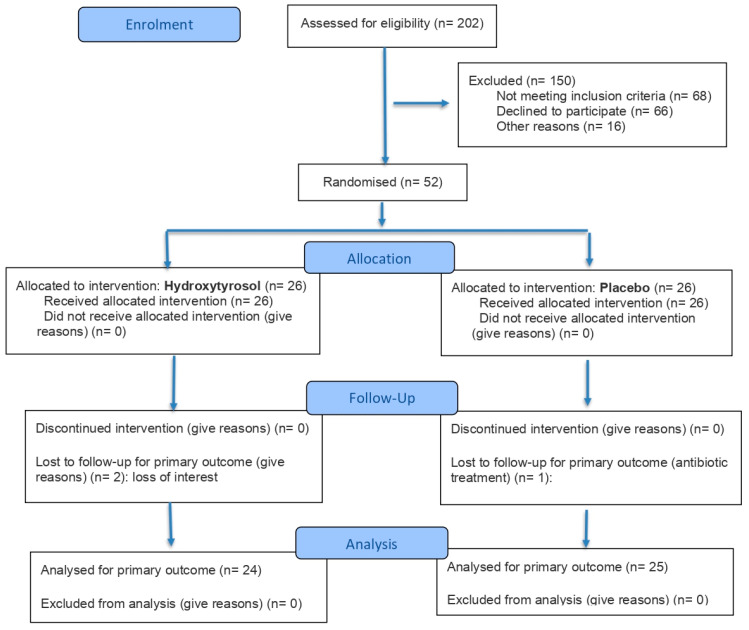
CONSORT 2025 flow diagram. Flow diagram of the progress through the phases of a randomised trial of two groups (that is, enrolment, intervention allocation, follow-up, and data analysis).

The clinical trial was conducted with 49 participants with overweight (BMI 25–30 kg/m^2^) and prediabetes (fasting plasma glucose 100–126 mg/dL and/or glycated hemoglobin [HbA1c] 5.7–6.4%), aged between 40 and 70 years. The CONSORT flow diagram is provided in Figure 1. Participants were allocated into two intervention groups: one group received a daily dose of 15 mg hydroxytyrosol (HT; n = 24), and the other received a placebo (P; n = 25), for 16 weeks. In addition, no adverse effects were recorded following the treatment in either group, and compliance was verified with changes in HT-3′-sulfate levels, increasing 0.25 µM (Confidence interval: −0.10; 0.63) in HT group and decreasing −0.12 µM (CI: −0.34; 0.10) in P group (*p =* 0.4) [12].

As shown in Table 1, participants in both groups were comparable at baseline in terms of age, sex distribution, BMI, fasting glucose, and HbA1c. Moreover, none of these parameters showed significant changes within or between groups after the intervention period.

### 3.2. Metabolic Profile Comparison

Once the relevant variables were analyzed according to the study inclusion criteria and no significant differences between the intervention groups were observed at baseline, an untargeted, LC-MS-based metabolomic analysis on serum samples was performed. Using the data obtained from this approach, a univariate analysis was conducted employing *t*-test to compare the rate of change of each identified metabolite between both groups. This analysis of individual metabolite variations revealed differences between groups within specific metabolic families. For clarity, the results in this section are divided into two categories: non-lipid metabolites and lipid metabolites.

#### 3.2.1. Non-Lipidic Metabolites

**Figure 2:**
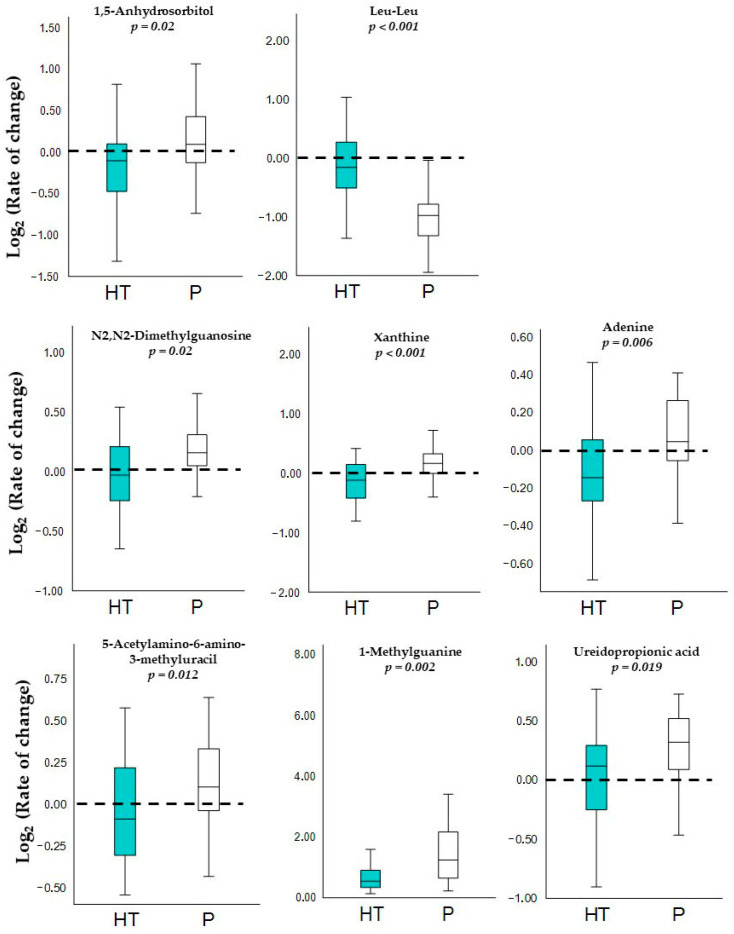
Boxplots show the log_2_ (rate of change) for the non-lipidic metabolites in the HT (N = 24) and P (N = 25) groups. Each panel includes the corresponding *p*-value obtained from unpaired Student’s *t*-test comparing both groups.

As shown in Table 2, targeted comparisons identified significant changes in non-lipidic metabolites belonging mainly to the carbohydrate, amino acid and peptide, as well as nucleotide and derivative families. In addition, these specific non-lipidic metabolite changes are illustrated by boxplot representations, in which all reported metabolites exhibited statistically significant differences (*p* < 0.05) between the HT and placebo groups (Figure 2).

First, circulating levels of 1,5-anhydrosorbitol were significantly reduced in participants receiving HT, whereas concentrations of the dipeptide Leu–Leu were increased compared with the placebo group. Regarding nucleotide derivatives, N_2_,N_2_-dimethylguanosine, 5-acetylamino-6-amino-3-methyluracil, 1-methylguanine, xanthine and ureidopropionic acid showed significantly lower concentrations in the HT group in comparison to the placebo group.

Overall, participants receiving HT displayed a consistent trend towards lower levels of metabolites related to nitrogenous base metabolism, supporting the idea of a selective effect of HT on this metabolic pathway rather than a broad metabolic remodeling.

#### 3.2.2. Lipid Metabolites

**Figure 3:**
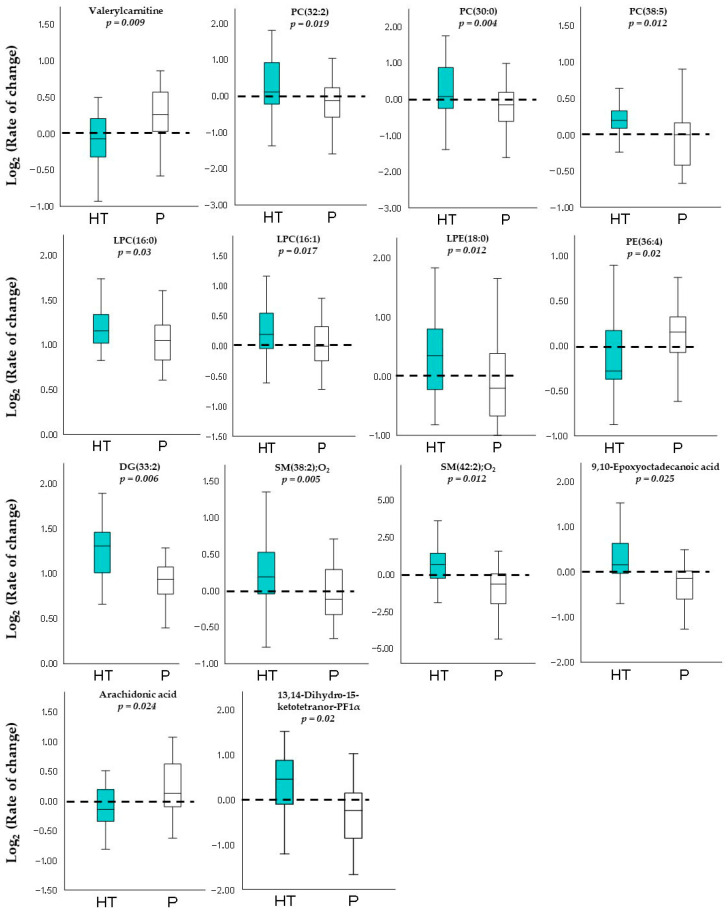
Boxplots show the log_2_ (rate of change) for the lipidic metabolites in the HT (N = 24) and P (N = 25) groups. Each panel includes the corresponding *p*-value obtained from unpaired -Student’s *t*-test comparing both groups. DG, diacylglycerol; LPC, lysophosphatidylcholine; LPE, lysophosphatidylethanolamine; PC, phosphatidylcholine; PE, phosphatidylethanolamine; SM, sphingomyelin.

Regarding lipids and their derivatives, participants receiving HT exhibited a general increase in several metabolites within this category, particularly lysophosphatidylcholines, phosphatidylcholines, lysophosphatidylethanolamines, diacylglycerols, sphingomyelins, fatty acids, and prostaglandin derivatives. In contrast, valerylcarnitine, PE(36:4), and arachidonic acid showed significantly decreased levels in the HT group compared with the placebo group (Table 3). These differences are further illustrated in the corresponding boxplots, where each lipid metabolite is accompanied by its respective *p*-value, as shown in Figure 3.

### 3.3. Multivariant Analysis

#### 3.3.1. Principal Component Analysis (PCA) of the Metabolites in the Two Groups

**Figure 4:**
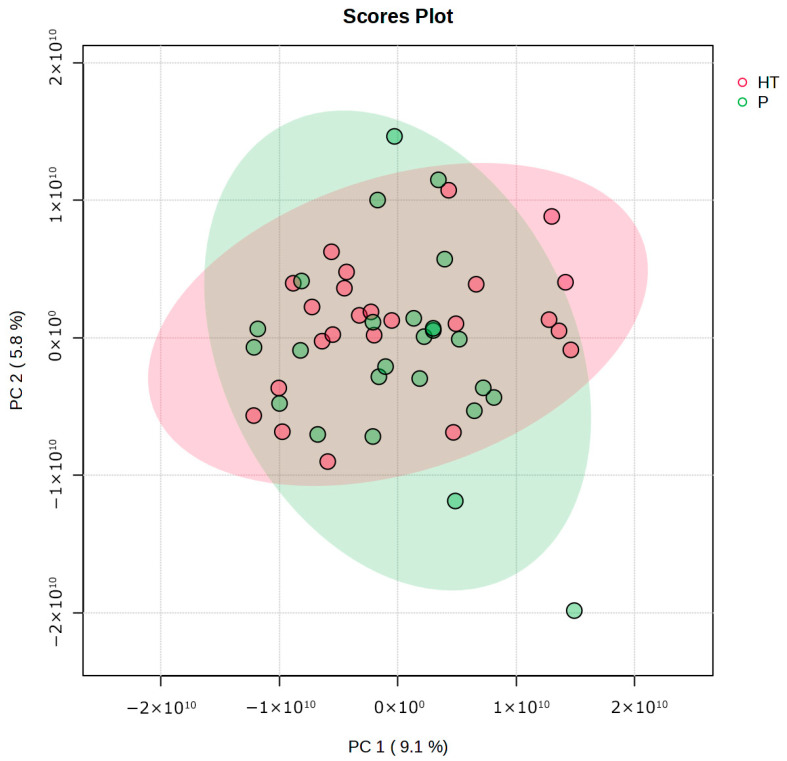
Principal component analysis (PCA) comparing metabolites between groups HT (red circles, N = 24) and P (green circles, N = 25). PC, principal component.

To obtain a preliminary overview of global differences in metabolite changes between intervention groups, as well as the degree of within-group variability, an exploratory PCA was performed on the entire dataset, including 878 metabolites identified (Figure 4). The principal components did not clearly separate the groups, as the corresponding clusters largely overlapped. This finding indicates that, although differences in specific metabolites may be present, they are not sufficiently pronounced to produce a clear global discrimination between groups attributable to the HT intervention. Moreover, the limited variance explained by the first two principal components (14.9% in total) indicates high multivariate dispersion and a complex underlying data structure.

#### 3.3.2. Hierarchical Clustering Heatmap and Variable Importance in Projection (VIP) Analysis

Univariate analysis identified a number of metabolites that differed significantly between groups using Student’s *t*-test. However, multivariate approaches such as VIP analysis and hierarchical clustering heatmaps allow the identification of additional metabolites that, although not reaching statistical significance individually, contribute to the overall separation between groups. These complementary analyses highlight specific metabolites that may be biologically relevant and improve interpretation of the metabolic pathways underlying the observed group differences.

**Figure 5:**
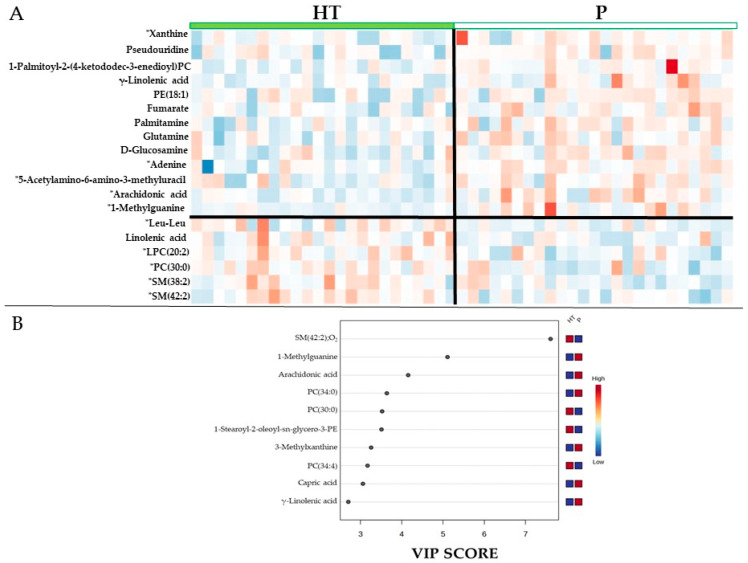
Heatmap and variable importance analysis of discriminant serum metabolites between intervention groups. (**A**) Heatmap showing the relative signal intensity (normalized abundance) of metabolites included in the hierarchical clustering across individual participants receiving hydroxytyrosol (HT, N = 24) or placebo (P, N = 25). Metabolites marked with an asterisk (*) indicate those that were statistically significant between groups, while non-significant metabolites are also shown to illustrate the overall clustering and visual separation between groups. Color scale represents relative abundance (red, higher; blue, lower). (**B**) Variable Importance in Projection (VIP) scores derived from the PLS-DA model, indicating the metabolites that most strongly contributed to discrimination between the HT and placebo groups. PC, phosphatidylcholine; LPC, lysophosphatidylcholine; SM, sphingomyelin; PE, phosphatidylethanolamine.

Figure 5A shows a heatmap of discriminant metabolites, revealing a clear clustering pattern between both intervention groups. Several metabolites exhibited lower relative abundance in the HT group compared with placebo, particularly those associated with purine and pyrimidine metabolism (xanthine, pseudouridine, glutamine, adenine, 5-acetylamino-6-amino-3-methyluracil, and 1-methylguanine) and eicosanoid biosynthesis (arachidonic acid and γ-linolenic acid), among others. In contrast, the dipeptide Leu–Leu, linolenic acid, phosphatidylcholines (LPC(20:2) and PC(30:0)), and sphingomyelins (SM(38:2);O2 and SM(42:2);O2) showed higher relative levels in the HT group. The contrasting color gradients highlight group-specific metabolic signatures, indicating marked differences in purine metabolism, lipid species, and amino acid–related intermediates.

To identify the metabolites contributing most strongly to group discrimination, a VIP analysis was performed (Figure 5B). Metabolites with the highest VIP scores included SM(42:2);O2, 1-methylguanine, arachidonic acid, PC(34:0), and PC(30:0), confirming their strong discriminative power. Additional contributors such as capric acid, γ-linolenic acid, 1-stearoyl-2-oleoyl-sn-glycero-3-PE (PE36:1;(18:0/18:1), PC(34:4) and 3-methylxanthine also exceeded the significance threshold. Collectively, these findings indicate that both lipid-derived compounds and purine-related metabolites are key drivers distinguishing the metabolic profiles of the HT and placebo groups.

### 3.4. Correlations Between Metabolites and Oxidative Status and Inflammation Parameters

**Figure 6:**
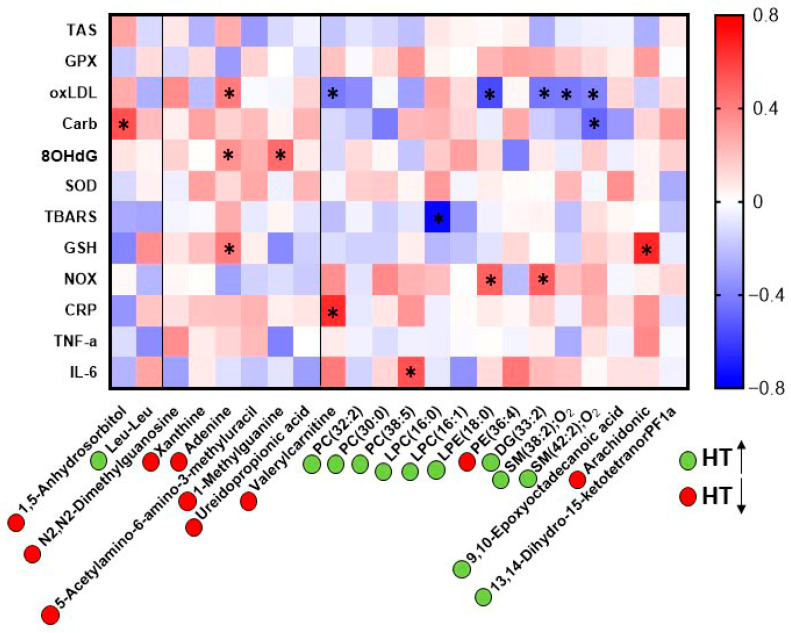
Correlation heatmap between significantly altered serum metabolites in the HT (N = 23) group and biochemical biomarkers related to oxidative stress, lipid peroxidation, and inflammation. Pearson correlation coefficients were used to generate the matrix. Red indicates positive correlations and blue indicates negative correlations, with color intensity reflecting the strength of the association. Metabolites marked in green were higher in HT group, whereas those in red were minor. Asterisks denote significant correlations (*p* < 0.05). Carb: Protein Carbonyls; CRP: C-Reactive Protein; GPX: Glutathione Peroxidase; GSH: Reduced Glutathione; IL-6: Interleukin-6; NOX: NADPH Oxidase activity; 8OHdG: 8-Hydroxy-2′-deoxyguanosine; oxLDL: Oxidized Low-Density Lipoprotein; SOD: Superoxide Dismutase; TAS: Total Antioxidant Status; TBARS: Thiobarbituric Acid Reactive Substances; TNF-α: Tumor Necrosis Factor alpha.

Next, we examined the relationships between the significant metabolites detected in the serum of the HT group and previously reported clinical parameters related to oxidative status, lipid peroxidation, and inflammation [12]. Pearson correlation analysis was performed, and the results were visualized in a heatmap, where red indicates a positive association and blue indicates a negative association (Figure 6).

Oxidized LDL (oxLDL) showed a significant negative correlation with several lipid species elevated in the HT-treated group, including SM(38:2);O_2_, SM(42:2);O_2_, DG(33:2), and LPE(18:0). In addition, valerylcarnitine, which was decreased in the HT group, also exhibited a negative correlation with oxLDL. On the contrary, oxLDL showed a significant positive correlation with adenine, which decreased after HT supplementation. Conversely, protein carbonyls displayed a positive correlation with 1,5-anhydrosorbitol, a metabolite that decreased following HT supplementation.

Regarding markers of DNA oxidative damage, 8-hydroxy-2′-deoxyguanosine (8-OHdG) was positively correlated with metabolites involved in nitrogen-base metabolism, particularly adenine and 1-methylguanine. For lipid oxidative stress, assessed by TBARS, a negative correlation was observed with LPC(16:0), which was increased in the HT group. Glutathione (GSH) correlated positively with adenine and arachidonic acid, both of which were reduced after HT supplementation. Additionally, NADPH oxidase (NOX) activity was positively correlated with LPE(18:0) and DG(33:2), two lipids elevated in the HT group. Inflammatory parameters also exhibited specific associations: C-reactive protein (CRP) correlated positively with valerylcarnitine, whereas interleukin-6 (IL-6) showed a positive correlation with PC(38:5).

Taken together, these significant associations suggest that HT supplementation may selectively modulate metabolites that interact with key pathways related to oxidative stress and inflammation.

## 4. Discussion

A LC–MS–based metabolomic analysis compared participants receiving 15 mg/day of HT with those receiving a placebo over a 16-week intervention period, during which HT had previously been shown to improve oxidative and inflammatory status [12]. The HT group exhibited lower levels of nitrogenous bases and related metabolites, together with higher concentrations of several lipid-related species (valerylcarnitine, lysophosphatidylcholines, phosphatidylcholines, and long-chain sphingomyelins) and reduced arachidonic acid compared with placebo group. Although causal relationships cannot be established in untargeted metabolomics, we propose biologically plausible mechanisms to interpret these associations.

While the EFSA recognizes a minimum daily intake of 5 mg of olive polyphenols, including HT, consumed as extra virgin olive oil to substantiate the associated health claim, the 15 mg/day dose used in this study (threefold higher) was selected to ensure observable effects on clinical parameters, particularly oxLDL. The dose range applied in previous intervention studies is broad (5–100 mg/day), with evidence indicating that measurable effects on health outcomes are observed from 15 mg/day onwards, especially in populations at elevated metabolic risk, such as in the present study [15]. Despite the considerable variability in the polyphenol content of olive oils, concentrations around 300 mg/kg are commonly reported [16] and given typical olive oil consumption recommendations of approximately 50 mL/day, an intake of 15 mg/day of HT could be readily achieved through a diet that includes extra virgin olive oil [17].

Regarding non-lipid (carbohydrates, peptides, nitrogenous bases and derivatives) metabolites, we found that the carbohydrate 1,5-anhydrosorbitol as reduced in the HT group. This marker reflects short-term glycemic peaks because it competes with glucose for renal reabsorption via SGLT2 [18]. Interestingly, polyphenol-rich foods and isolated polyphenols have been shown to inhibit SGLT2 [19,20], offering a plausible alternative explanation for this decrease. On the other hand, although elevated circulating branched-chain amino acids are typically linked to insulin resistance [21], the dipeptide Leu–Leu was increased in the HT group. This is consistent with evidence showing that several dipeptides (including Ile–Ile/Leu–Leu) are reduced in women with morbid obesity and T2D compared with normal-weight controls [22], suggesting that certain dipeptides may reflect a healthier metabolic phenotype, although their biological role remains poorly understood.

Notably, the most robust signal among non-lipid metabolites was a reduction in nitrogenous base derivatives in the HT group. This aligns with the protective role of HT, as increased oxidative DNA damage and consequent nucleotide turnover have been reported in metabolic diseases [23]. Two nucleoside-derived metabolites, N_2_, N_2_-dimethylguanosine and pseudouridine, both associated with reduced longevity [24], were diminished in the HT group in comparison to placebo. Likewise, lower levels of ureidopropionic acid and xanthine in patients treated with HT suggest less activation of pyrimidine and purine catabolism, respectively. Meanwhile, increased glutamine retained in the multivariate analysis supports the activation of nucleotide biosynthetic pathways in the placebo group. These findings are consistent with recent reports of elevated adenine in individuals with T2D [25], potentially linked to altered ATP metabolism in insulin resistance. In line with our results, other authors [11] observed lower hypoxanthine, xanthine and uridine in participants responding to higher-dose HT supplementation, while adenine decreased in those individuals. Nevertheless, nitrogen metabolism varies considerably depending on the severity of the insulin resistance [26,27], which might explain the differences in adenine levels between studies.

Beyond its antioxidant effects on nucleic acids, these findings suggest a potential modulation of purine metabolism, possibly involving xanthine oxidase, an enzyme upregulated in obesity. In other oxidative stress contexts such as exercise, polyphenol-rich diets attenuate the accumulation of purine degradation products and their association with oxidative stress markers [28]. Although some studies report no inhibitory effect of HT on xanthine oxidase [29], experimental models of hyperuricemia support a potential inhibitory role for HT [30]. Such inhibition could reduce the irreversible purine degradation to uric acid and favor salvage pathways, providing a coherent mechanistic explanation for the lower adenine and xanthine levels observed after HT supplementation in comparison to placebo.

Turning to lipid metabolites, obesity is characterized by chronic free fatty acid oversupply, which can overwhelm mitochondrial β-oxidation and leads to the accumulation of intermediates such as acylcarnitines and diacylglycerols, together with a relative deficiency of free carnitine. This imbalance can induce mitochondrial stress and lipotoxic effects, as carnitine normally buffers potentially toxic acyl-CoA species within mitochondria [31]. The resulting accumulation of circulating acylcarnitines has been associated with T2D [32], highlighting how disrupted lipid handling contributes to the metabolic dysfunction. In the present study, however, valerylcarnitine was reduced and DG(33:2) was increased in the HT group, which limits the strength of any mechanistic interpretation linking HT supplementation to improved fatty acid oxidation.

On the other hand, phospholipids are essential structural components of cell membranes, and alterations in their profiles have been linked to a wide range of diseases, supporting their potential utility as biomarkers [33]. Participants receiving HT exhibited higher levels of several phosphatidylcholines (PC 30:0, PC 32:2, PC 38:5) and lysophosphatidylcholines (LPC 16:0, LPC 16:1) compared with placebo group. In agreement with our findings, Paapstel et al., [34] reported higher circulating levels of PCs and LPCs, including PC 30:0 and PC 32:2, in healthy individuals compared with those with atherogenic profiles. Conversely, other study has reported elevated LPC and LPE levels in women with morbid obesity relative to metabolically healthy individuals, underscoring the context-dependent nature of phospholipid alterations [22].

Regarding sphingomyelins, two species were increased in the HT group, with SM(42:2);O_2_ ranking first in the VIP analysis. Previous studies have shown that low circulating levels of long-chain unsaturated sphingomyelins, such as SM(38:1), SM(41:1) and SM(42:1), predict higher mortality risk [35]. Nevertheless, their role in metabolic diseases remains incompletely understood, as some species have been associated with increased risk of T2D, whereas others appear to be protective [32].

The most striking lipid-related change was the reduction in arachidonic acid in the HT group compared with placebo, given its central role as a precursor of pro-inflammatory eicosanoids. Multivariate analysis further revealed lower gamma-linolenic acid and higher linoleic acid, the upstream precursor of this pathway. These findings are consistent with preclinical metabolomic studies showing that HT supplementation downregulates arachidonic acid metabolism in mice with metabolic syndrome, resulting in reduced prostaglandin release and attenuation of chronic low-grade inflammation [36]. Moreover, HT has been reported to inhibit key enzymes in the arachidonic acid cascade, including phospholipase A2, cyclooxygenases and lipoxygenases [37,38]. This event, together with the known anti-inflammatory potential of HT, supports a dual mechanistic hypothesis: reduced mobilization of arachidonic acid from membrane phospholipids via phospholipase A2 inhibition, and/or attenuation of desaturase activity, whose expression is upregulated by inflammatory stimuli. The net effect would be preservation of linoleic acid and reduced conversion to arachidonic acid, thereby contributing to the lower inflammatory status observed in individuals receiving HT supplementation.

The remaining metabolites, 9,10-epoxyoctadecanoic acid and 13,14-dihydro-15-ketotetranor-PGF1α, were also elevated in the HT group, although their biological significance remains uncertain due to the lack of clinical evidence [39].

Moreover, variability in the response to polyphenols between individuals should be considered, as factors such as sex, gut microbiota composition and specific allelic variants of hepatic enzymes can influence the potential health effects of these compounds [40]. Although stratified analyses according to these variables were not performed in the present study, this was due to the limited sample size. Subdivision into multiple subgroups would have resulted in insufficient numbers of participants per category to provide representative or statistically robust estimates. Nevertheless, future research should account for these factors to help identify response patterns that may otherwise remain undetected.

Finally, the correlations observed between significant metabolites and markers of oxidative stress and inflammation revealed statistically significant associations, although only a limited number showed correlation coefficients above 0.5. The most noteworthy findings were the positive correlations between adenine and 1-methylguanine with 8-OHdG, which may reflect a link between oxidative DNA damage and activation of nucleotide metabolic pathways [23]. In addition, several lipid species that were increased in the HT group exhibited negative correlations with oxLDL, suggesting that the presence of specific lipid species may be associated with reduced oxidative modification of circulating LDL particles.

## 5. Conclusions

**Figure 7:**
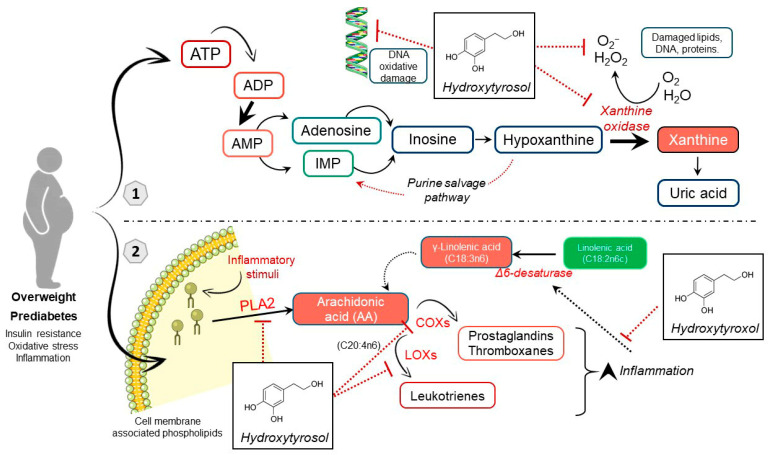
Proposed mechanistic pathways underlying the metabolic effects of hydroxytyrosol in individuals with overweight and prediabetes. Hydroxytyrosol (HT) supplementation may modulate two key metabolic pathways. (1) Purine metabolism: HT is hypothesized to attenuate oxidative stress and reduce oxidative DNA damage, thereby lowering nucleotide turnover. A potential inhibitory effect on xanthine oxidase (XO) could decrease the conversion of hypoxanthine to xanthine and uric acid, while favoring purine salvage pathways, such as the formation of inosine monophosphate (IMP). (2) Arachidonic acid (AA) metabolism: HT may reduce the mobilization of AA from membrane phospholipids by inhibiting phospholipase A2 (PLA2) and may downregulate downstream inflammatory pathways through inhibition of cyclooxygenases (COXs) and lipoxygenases (LOXs), leading to reduced production of pro-inflammatory eicosanoids, including prostaglandins, thromboxanes and leukotrienes. In addition, HT may influence fatty acid desaturation through modulation of Δ6-desaturase activity, contributing to altered levels of linoleic acid (LA) and γ-linolenic acid (GLA). Collectively, these mechanisms may help explain the observed reductions in oxidative stress and inflammation. Compounds shown in red indicate decreased levels in the HT group, whereas compounds shown in green indicate increased levels. Solid black arrows indicate direct transformations, dotted arrows indicate the presence of intermediates, and red dotted flat-headed arrows indicate inhibition.

This study demonstrates that HT supplementation (15 mg/day for 16 weeks) induces changes in the serum metabolic profile of individuals with prediabetes and overweight. Untargeted metabolomics enabled the identification of potential pathways through which HT may modulate endogenous metabolism, providing mechanistic insights into its effects following chronic intake. Overall, the metabolic differences between the HT and placebo groups point to two main pathways potentially involved: purine degradation and arachidonic acid metabolism (Figure 7).

HT supplementation was associated with reduced serum levels of nitrogenous base derivatives and arachidonic acid, together with increased concentrations of phosphatidylcholines, lysophosphatidylcholines and sphingomyelins. It is plausible that HT attenuates oxidative damage, thereby reducing the activation of nucleotide biosynthesis and degradation pathways, which, together with inhibitory effects on xanthine oxidase activity, would lower the production of purine degradation products such as xanthine and consequently reduce oxidative stress. In parallel, the improvement in inflammatory status and oxidative stress attributed to HT [12] may be explained by decreased synthesis of arachidonic acid and/or reduced mobilization from membrane phospholipids, ultimately resulting in lower production of pro-inflammatory prostaglandins.

These findings should be considered as exploratory and require confirmation in larger clinical studies. Future trials should integrate metabolomic analyses to better characterize how polyphenols modulate metabolic pathways, ideally in combination with other omics approaches such as metagenomics, transcriptomics and genomics, to enable more robust and interconnected biological interpretations, and to assess whether these metabolic signatures are consistently observed in other populations at cardiometabolic risk.

## Figures and Tables

**Table 1:** Characteristics of hydroxytyrosol (HT) and placebo (P) groups pre-intervention (Pre-int) and post-intervention (Post-int). Values are presented as means ± standard deviations. Groups were compared with χ^2^ statistics for sex and *t*-tests for continuous variables. BMI (body mass index), HbA1c (glycated hemoglobin).

	HT (N = 24)		P (N = 25)	
	Pre-Int	Post-Int	Pre-Int	Post-Int
Age (years)	54.50 ± 8.55		57.40 ± 7.90	
Sex (% female)	45.83		44.00	
Weight (kg)	80.55 ± 9.57	80.62 ± 9.84	78.92 ± 9.76	79.25 ± 9.90
BMI (kg/m^2^)	29.17 ± 2.86	28.77 ± 2.69	27.94 ± 2.69	28.18 ± 2.65
Fasting glucose (mg/dL)	97.50 ± 10.11	99.52 ± 10.54	93.08 ± 6.94	98.92 ± 8.70
HbA1c (%)	5.80 ± 0.31	5.87 ± 0.31	5.78 ± 0.29	5.85 ± 0.25

**Table 2:** Non-lipidic metabolite rate of changes in serum samples of HT treated participants (N = 24) compared to placebo treated participants (N = 25).

Group	Class	Increased Metabolite Levels in HT	Decreased Metabolite Levels in HT
Carbohydrates	Polyols		1,5-Anhydrosorbitol
Amino acids and peptides	Peptides	Leu-Leu	
Nucleotides and derivates	Nucleoside		N2,N2-Dimethylguanosine
	Nitrogenous bases		Xanthine Adenine 5-Acetylamino-6-amino-3-methyluracil 1-Methylguanine
	Nitrogenous bases derivates		Ureidopropionic acid

**Table 3:** Lipid metabolites rate of change in serum samples of HT treated participants (N = 24) compared to placebo treated participants (N = 25).

Group	Class	Increased Metabolite Levels in HT	Decreased Metabolite Levels in HT
Lipids and derivates	Acylcarnitines		Valerylcarnitine
	Lysophosphatidylcholine (LPC) and phosphatidylcholine (PC)	LPC(16:0) LPC(16:1) PC(32:2) PC(30:0) PC(38:5)	
	Lysophosphatidylethanolamine (LPE) and phosphatidylethanolamine (PE)	LPE(18:0)	PE(36:4)
	Diacylglycerol (DG)	DG(33:2)	
	Sphingomyelins (SM)	SM(38:2);O2 SM(42:2);O2	
	Fatty acids	9,10-Epoxyoctadecanoic acid	Arachidonic acid
	Prostaglandins	13,14-Dihydro-15-ketotetranorPF1α	

## Data Availability

The data for this article, including all the raw data used in the generation of tables and figures (also those provided as ESI†), are available at the public repository digital.csic.es of the Spanish Research Council (CSIC) at https://doi.org/10.20350/digitalCSIC/17951.
